# Changing epidemiology and trends in incidence of thyroid cancer in England, 1985-2019

**DOI:** 10.1093/eurpub/ckac130.053

**Published:** 2022-10-25

**Authors:** R Stonell, P Bannister, A Memon

**Affiliations:** Brighton and Sussex Medical School, Department of Primary Care and Public Health, Brighton, UK

## Abstract

**Background:**

Thyroid cancer is 2-3 times more common in females and is currently the fastest growing cancer worldwide. Exposure to ionizing radiation is the only established risk factor for thyroid cancer. Other factors include obesity, history of benign thyroid conditions, and family history. We conducted a retrospective population-based cohort study to examine whether there have been changes in the incidence of thyroid cancer in England during the past four decades.

**Methods:**

Individual level data for patients diagnosed with thyroid cancer in England during 1985-2019 were obtained from the Office for National Statistics/Public Health England. Average annual incidence rates were calculated by two age categories (0-49, 50+ years) and all ages combined during the seven five-year time periods (1985-89 to 2015-19). The percentage change in incidence was calculated as change in the average annual incidence rate from the first (1985-89) to the last time period (2015-19).

**Results:**

During the 35-year study period, a total of 58,710 new cases of thyroid cancer were registered in England (27.3% males, 72.7% females). In young people aged 0-49 years, the average annual incidence rates increased by 375% in males and 438% in females (from 0.4/100,000 in 1985-89 to 1.9/100,000 in 2015-19 in males and from 1.3/100,000 in 1985-89 to 7.0/100,000 in 2015-19 in females). In older people aged 50+ years, the rates increased by 146% in males and 171% in females (from 2.4/100,000 in 1985-89 to 5.9/100,000 in 2015-19 in males and from 4.1/100,000 in 1985-89 to 11.1/100,000 in 2015-19 in females).

**Conclusions:**

There has been a steady and substantial increase in the incidence of thyroid cancer in England over the past four decades. The largest increase in incidence was observed in young people aged 0-49 years. Some of this increase is due to enhanced surveillance and sensitive diagnostic methods, but other factors (e.g., obesity and history of benign thyroid conditions) need to be considered.

**Key messages:**

- The unexpected substantial increase in the incidence of thyroid cancer in young people aged 0-49 years needs further investigation.
- It is important to determine what proportion of the cases are due to enhanced surveillance and what proportion are due to environmental exposures.

